# Bis­(2-methyl-1*H*-imidazole-κ*N* ^3^)bis[2-(naphthalen-2-yl)acetato-κ*O*]copper(II)

**DOI:** 10.1107/S1600536811047969

**Published:** 2011-11-16

**Authors:** Fu-Jun Yin, Li-Jun Han, Shu-Ping Yang, Xing You Xu

**Affiliations:** a Jiangsu Marine Resources Development Research Institute, Huaihai Institute of Technology, Lianyungang 222005, People’s Republic of China; b Department of Mathematics and Science, Huaihai Institute of Technology, Lianyungang 222005, People’s Republic of China; c Department of Chemical Engineering, Huaihai Institute of Technology, Lianyungang 222005, People’s Republic of China; d Huaiyin Insititute of Technology, Huaiyin 223003, People’s Republic of China

## Abstract

In the crystal structure of the title compound, [Cu(C_12_H_9_O_2_)_2_(C_4_H_6_N_2_)_2_], the Cu(II) cations are square-planar coordinated by two 1-naphthyl­acetate anions and two 2-methyl-imidazole ligands into discrete complexes that are located on centres of inversion. These complexes are linked into chains parallel to [010] by inter­molecular N—H⋯O hydrogen bonding between the N—H H atom of the 2-methyl-imidazole ligands and the carboxyl­ate O atoms that are not involved in metal coordination.

## Related literature

For related structures, see: Liu *et al.* (2007 ▶); Chen *et al.* (2004 ▶); Yang *et al.* (2008 ▶); Tang *et al.* (2006 ▶); Ji *et al.* (2011 ▶). 
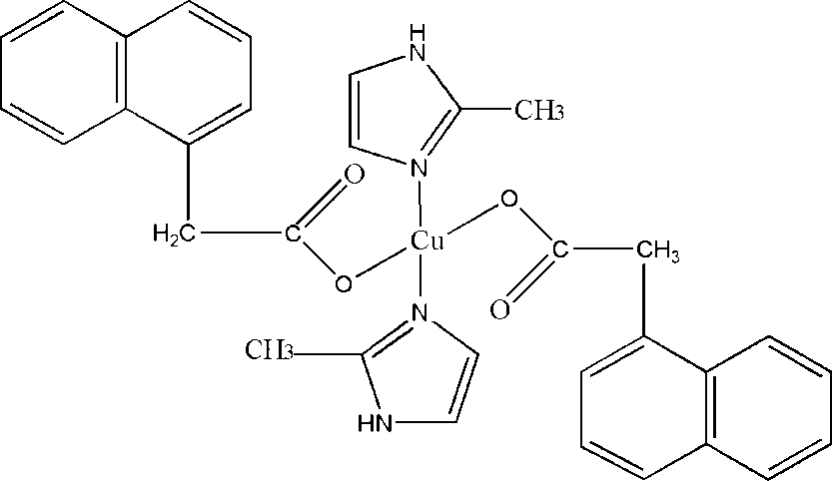


## Experimental

### 

#### Crystal data

[Cu(C_12_H_9_O_2_)_2_(C_4_H_6_N_2_)_2_]
*M* *_r_* = 598.14
Monoclinic, 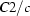

*a* = 28.430 (4) Å
*b* = 7.5544 (10) Å
*c* = 13.9417 (19) Å
β = 108.216 (2)°
*V* = 2844.2 (7) Å^3^
*Z* = 4
Mo *K*α radiation
μ = 0.81 mm^−1^
*T* = 298 K
0.12 × 0.10 × 0.10 mm

#### Data collection

Bruker APEXII CCD diffractometer
Absorption correction: multi-scan (*SADABS*; Sheldrick, 1996 ▶) *T*_min_ = 0.866, *T*_max_ = 0.903
10438 measured reflections
2507 independent reflections
2081 reflections with *I* > 2σ(*I*)
*R*_int_ = 0.032

#### Refinement

*R*[*F* ^2^ > 2σ(*F* ^2^)] = 0.034
*wR*(*F* ^2^) = 0.090
*S* = 1.05
2507 reflections
188 parameters
H-atom parameters constrained
Δρ_max_ = 0.23 e Å^−3^
Δρ_min_ = −0.22 e Å^−3^

### 

Data collection: *APEX2* (Bruker, 2007 ▶); cell refinement: *SAINT* (Bruker, 2007 ▶); data reduction: *SAINT*; program(s) used to solve structure: *SHELXS97* (Sheldrick, 2008 ▶); program(s) used to refine structure: *SHELXL97* (Sheldrick, 2008 ▶); molecular graphics: *SHELXTL* (Sheldrick, 2008 ▶); software used to prepare material for publication: *SHELXTL*.

## Supplementary Material

Crystal structure: contains datablock(s) I, global. DOI: 10.1107/S1600536811047969/nc2255sup1.cif

Structure factors: contains datablock(s) I. DOI: 10.1107/S1600536811047969/nc2255Isup2.hkl

Additional supplementary materials:  crystallographic information; 3D view; checkCIF report

## Figures and Tables

**Table 1:** Hydrogen-bond geometry (Å, °)

*D*—H⋯*A*	*D*—H	H⋯*A*	*D*⋯*A*	*D*—H⋯*A*
N2—H2⋯O2^i^	0.86	1.97	2.775 (3)	155

Symmetry code: (i) 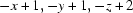.

**Fig. 1.**
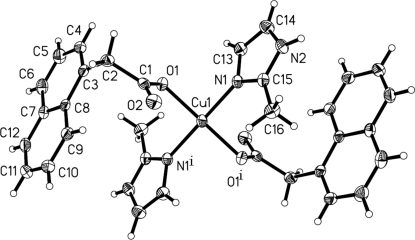
Crystal structure of the title compound with labeling and displacement ellipsoids drawn at the 30% probability level. Symmetry code: i= -x + 1, -y, -z + 2)

**Fig. 2.**
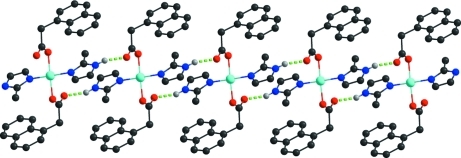
Part of the crystal structure of the title compound. Hydrogen bonding is shown as dashed lines and H atoms not involved in hydrogen bonding are omitted for clarity.

## supplementary crystallographic information

### Comment

The crystal structure of the title compound was determined as part of an ongoing study on the structural properties of copper complexes containing imidazole ligands. In this study 1-naphthylacetate was used as ligand, for which only a few metal complexes were reported (Liu *et al.*,2007; Chen *et al.*, 2004; Yang *et al.*, 2008; Tang *et al.*,2006 ; Ji *et al.*,2011).

In the crystal structure of the title compound [Cu(C_12_H_9_O_2_)_2_(C_4_H_6_N_2_)_2_], each copper cation are coordinated by two N atoms of two symmetry equivalent 2-methyl-1*H*-imidazole ligands and by two carboxyl O atoms of two symmetry related 1-naphthylacetate anions within a square planar coordination (Fig. 1). The Cu—N and Cu—O bond lengths are 1.9750 (18) and 1.9791 (16) Å, respectively. The asymmetric unit consits of one Cu(II) cation that is located on a center of inversion as well of one neutral and one anionic ligand in general positions. The discrete complexes are linked into chains by N—H···O hydrogen bonding (Fig. 2 and Table 1).

### Experimental

The title compound was synthesized by the reaction of Cu(NO_3_)_2_.3 H_2_O (72.3 mg, 0.3 mmol), 1-naphthylacetic acid (93 mg, 0.5 mmol), 2-methylimidazole (32.8 mg, 0.4 mmol) and NaOH (20 mg, 0.5 mmol) in 4 mL of a water-ethanol mixture (volume ratio = 1:1 of water:ethanol) under solvothermal conditions. The starting mixture was homogenized and transferred into a sealed teflon-lined solvothermal bomb (volume: 25 ml) and heated at 140° for three days. After cooling green crystals of the title compound were obtained, which were washed with distilled water and absolute ethyl alcohol (yield: 44.6% based on Cu(NO_3_)_2_.3 H_2_O ).

### Refinement

The C-H H atoms were positioned with idealized geometry (methyl H atoms allowed to rotatae but not to tip) and were refined with *U*_iso_(H) =1.2*U*_eq_(C)] (1.5 for methyl H atoms) using a riding model.

### Crystal data

**Table:**

[Cu(C_12_H_9_O_2_)_2_(C_4_H_6_N_2_)_2_]	*F*(000) = 1244
*M**_r_* = 598.14	*D*_x_ = 1.397 Mg m^−^^3^
Monoclinic, *C*2/*c*	Mo *K*α radiation, λ = 0.71073 Å
Hall symbol: -C 2yc	Cell parameters from 2712 reflections
*a* = 28.430 (4) Å	θ = 2.8–22.8°
*b* = 7.5544 (10) Å	µ = 0.81 mm^−^^1^
*c* = 13.9417 (19) Å	*T* = 298 K
β = 108.216 (2)°	Block, green
*V* = 2844.2 (7) Å^3^	0.12 × 0.10 × 0.10 mm
*Z* = 4	

### Data collection

**Table:**

Bruker APEXII CCD diffractometer	2507 independent reflections
Radiation source: fine-focus sealed tube	2081 reflections with *I* > 2σ(*I*)
graphite	*R*_int_ = 0.032
φ and ω scans	θ_max_ = 25.0°, θ_min_ = 2.8°
Absorption correction: multi-scan (*SADABS*; Sheldrick, 1996)	*h* = −33→33
*T*_min_ = 0.866, *T*_max_ = 0.903	*k* = −8→8
10438 measured reflections	*l* = −16→16

### Refinement

**Table:**

Refinement on *F*^2^	Primary atom site location: structure-invariant direct methods
Least-squares matrix: full	Secondary atom site location: difference Fourier map
*R*[*F*^2^ > 2σ(*F*^2^)] = 0.034	Hydrogen site location: inferred from neighbouring sites
*wR*(*F*^2^) = 0.090	H-atom parameters constrained
*S* = 1.05	*w* = 1/[σ^2^(*F*_o_^2^) + (0.0424*P*)^2^ + 2.2367*P*] where *P* = (*F*_o_^2^ + 2*F*_c_^2^)/3
2507 reflections	(Δ/σ)_max_ < 0.001
188 parameters	Δρ_max_ = 0.23 e Å^−^^3^
0 restraints	Δρ_min_ = −0.22 e Å^−^^3^

### Special details

**Table:**

Geometry. All e.s.d.'s (except the e.s.d. in the dihedral angle between two l.s. planes) are estimated using the full covariance matrix. The cell e.s.d.'s are taken into account individually in the estimation of e.s.d.'s in distances, angles and torsion angles; correlations between e.s.d.'s in cell parameters are only used when they are defined by crystal symmetry. An approximate (isotropic) treatment of cell e.s.d.'s is used for estimating e.s.d.'s involving l.s. planes.
Refinement. Refinement of *F*^2^ against ALL reflections. The weighted *R*-factor *wR* and goodness of fit *S* are based on *F*^2^, conventional *R*-factors *R* are based on *F*, with *F* set to zero for negative *F*^2^. The threshold expression of *F*^2^ > σ(*F*^2^) is used only for calculating *R*-factors(gt) *etc*. and is not relevant to the choice of reflections for refinement. *R*-factors based on *F*^2^ are statistically about twice as large as those based on *F*, and *R*- factors based on ALL data will be even larger.

### Fractional atomic coordinates and isotropic or equivalent isotropic displacement parameters (Å^2^)

**Table:**

	*x*	*y*	*z*	*U*_iso_*/*U*_eq_	
C1	0.41432 (9)	0.1140 (3)	0.87770 (18)	0.0393 (5)	
C2	0.36810 (9)	0.1271 (3)	0.78610 (19)	0.0444 (6)	
H2A	0.3421	0.1850	0.8059	0.053*	
H2B	0.3754	0.2004	0.7354	0.053*	
C3	0.34946 (8)	−0.0505 (3)	0.74014 (17)	0.0387 (5)	
C4	0.35305 (9)	−0.0972 (4)	0.64815 (18)	0.0488 (6)	
H4	0.3677	−0.0190	0.6144	0.059*	
C5	0.33521 (10)	−0.2605 (4)	0.6031 (2)	0.0586 (7)	
H5	0.3384	−0.2895	0.5406	0.070*	
C6	0.31340 (10)	−0.3760 (4)	0.6506 (2)	0.0553 (7)	
H6	0.3011	−0.4829	0.6198	0.066*	
C7	0.30908 (8)	−0.3357 (3)	0.74649 (18)	0.0414 (6)	
C8	0.32775 (8)	−0.1709 (3)	0.79265 (16)	0.0356 (5)	
C9	0.32324 (9)	−0.1349 (3)	0.88921 (18)	0.0442 (6)	
H9	0.3357	−0.0293	0.9216	0.053*	
C10	0.30110 (10)	−0.2521 (4)	0.9353 (2)	0.0561 (7)	
H10	0.2985	−0.2253	0.9985	0.067*	
C11	0.28210 (10)	−0.4129 (4)	0.8889 (2)	0.0587 (7)	
H11	0.2666	−0.4911	0.9208	0.070*	
C12	0.28645 (9)	−0.4541 (4)	0.7972 (2)	0.0521 (7)	
H12	0.2743	−0.5618	0.7673	0.062*	
C13	0.54765 (9)	0.2344 (3)	0.88513 (18)	0.0453 (6)	
H13	0.5409	0.1555	0.8313	0.054*	
C14	0.57019 (10)	0.3915 (3)	0.8895 (2)	0.0509 (7)	
H14	0.5817	0.4416	0.8401	0.061*	
C15	0.55191 (8)	0.3503 (3)	1.02945 (18)	0.0381 (5)	
C16	0.54798 (10)	0.3857 (3)	1.13113 (19)	0.0500 (6)	
H16A	0.5212	0.3179	1.1404	0.075*	
H16B	0.5419	0.5095	1.1374	0.075*	
H16C	0.5784	0.3527	1.1815	0.075*	
N1	0.53589 (7)	0.2080 (2)	0.97253 (14)	0.0378 (5)	
N2	0.57289 (8)	0.4629 (3)	0.98082 (16)	0.0453 (5)	
H2	0.5859	0.5633	1.0036	0.054*	
O1	0.44990 (6)	0.0258 (2)	0.86540 (12)	0.0421 (4)	
O2	0.41557 (7)	0.1856 (2)	0.95858 (13)	0.0522 (5)	
Cu1	0.5000	0.0000	1.0000	0.03683 (15)	

### Atomic displacement parameters (Å^2^)

**Table:**

	*U*^11^	*U*^22^	*U*^33^	*U*^12^	*U*^13^	*U*^23^
C1	0.0456 (14)	0.0263 (11)	0.0454 (14)	−0.0092 (10)	0.0134 (11)	0.0017 (10)
C2	0.0460 (14)	0.0365 (13)	0.0504 (15)	0.0049 (11)	0.0146 (12)	0.0092 (11)
C3	0.0285 (12)	0.0464 (14)	0.0374 (13)	0.0043 (10)	0.0051 (10)	0.0046 (10)
C4	0.0409 (14)	0.0658 (17)	0.0397 (14)	−0.0029 (13)	0.0125 (11)	0.0072 (13)
C5	0.0520 (16)	0.082 (2)	0.0439 (15)	−0.0064 (15)	0.0177 (13)	−0.0145 (15)
C6	0.0461 (15)	0.0595 (17)	0.0565 (17)	−0.0061 (13)	0.0106 (13)	−0.0200 (14)
C7	0.0299 (12)	0.0470 (14)	0.0442 (14)	−0.0005 (10)	0.0072 (10)	−0.0022 (11)
C8	0.0250 (11)	0.0424 (13)	0.0361 (12)	0.0044 (9)	0.0045 (9)	0.0026 (10)
C9	0.0411 (14)	0.0503 (15)	0.0392 (13)	−0.0011 (11)	0.0098 (11)	−0.0009 (11)
C10	0.0477 (15)	0.080 (2)	0.0413 (15)	−0.0057 (14)	0.0151 (12)	0.0059 (14)
C11	0.0431 (15)	0.0704 (19)	0.0611 (18)	−0.0101 (14)	0.0143 (13)	0.0161 (16)
C12	0.0367 (14)	0.0507 (16)	0.0617 (18)	−0.0076 (11)	0.0052 (12)	0.0022 (13)
C13	0.0504 (15)	0.0453 (14)	0.0417 (14)	−0.0035 (12)	0.0167 (12)	−0.0097 (11)
C14	0.0587 (17)	0.0489 (15)	0.0517 (16)	−0.0046 (13)	0.0266 (13)	−0.0030 (12)
C15	0.0334 (12)	0.0346 (12)	0.0441 (13)	0.0019 (10)	0.0090 (10)	−0.0045 (10)
C16	0.0597 (16)	0.0451 (15)	0.0454 (15)	0.0053 (12)	0.0169 (13)	−0.0084 (12)
N1	0.0364 (11)	0.0340 (10)	0.0414 (11)	−0.0033 (8)	0.0098 (9)	−0.0055 (9)
N2	0.0486 (12)	0.0337 (11)	0.0555 (13)	−0.0086 (9)	0.0190 (10)	−0.0074 (9)
O1	0.0368 (9)	0.0444 (10)	0.0412 (9)	−0.0025 (7)	0.0065 (7)	−0.0054 (7)
O2	0.0678 (12)	0.0392 (9)	0.0518 (11)	−0.0075 (8)	0.0219 (9)	−0.0115 (8)
Cu1	0.0348 (2)	0.0341 (2)	0.0391 (2)	−0.00479 (17)	0.00786 (17)	−0.00590 (17)

### Geometric parameters (Å, °)

**Table:**

C1—O2	1.241 (3)	C10—H10	0.9300
C1—O1	1.267 (3)	C11—C12	1.358 (4)
C1—C2	1.523 (3)	C11—H11	0.9300
C2—C3	1.510 (3)	C12—H12	0.9300
C2—H2A	0.9700	C13—C14	1.341 (3)
C2—H2B	0.9700	C13—N1	1.375 (3)
C3—C4	1.364 (3)	C13—H13	0.9300
C3—C8	1.424 (3)	C14—N2	1.363 (3)
C4—C5	1.405 (4)	C14—H14	0.9300
C4—H4	0.9300	C15—N1	1.329 (3)
C5—C6	1.358 (4)	C15—N2	1.339 (3)
C5—H5	0.9300	C15—C16	1.481 (3)
C6—C7	1.413 (3)	C16—H16A	0.9600
C6—H6	0.9300	C16—H16B	0.9600
C7—C12	1.413 (3)	C16—H16C	0.9600
C7—C8	1.425 (3)	N1—Cu1	1.9750 (18)
C8—C9	1.418 (3)	N2—H2	0.8600
C9—C10	1.358 (3)	O1—Cu1	1.9791 (16)
C9—H9	0.9300	Cu1—N1^i^	1.9750 (18)
C10—C11	1.402 (4)	Cu1—O1^i^	1.9791 (16)
			
O2—C1—O1	123.7 (2)	C12—C11—H11	120.1
O2—C1—C2	120.4 (2)	C10—C11—H11	120.1
O1—C1—C2	115.9 (2)	C11—C12—C7	121.0 (3)
C3—C2—C1	113.22 (18)	C11—C12—H12	119.5
C3—C2—H2A	108.9	C7—C12—H12	119.5
C1—C2—H2A	108.9	C14—C13—N1	109.5 (2)
C3—C2—H2B	108.9	C14—C13—H13	125.3
C1—C2—H2B	108.9	N1—C13—H13	125.2
H2A—C2—H2B	107.7	C13—C14—N2	106.2 (2)
C4—C3—C8	119.3 (2)	C13—C14—H14	126.9
C4—C3—C2	120.6 (2)	N2—C14—H14	126.9
C8—C3—C2	120.1 (2)	N1—C15—N2	109.6 (2)
C3—C4—C5	121.6 (2)	N1—C15—C16	127.1 (2)
C3—C4—H4	119.2	N2—C15—C16	123.3 (2)
C5—C4—H4	119.2	C15—C16—H16A	109.5
C6—C5—C4	120.2 (2)	C15—C16—H16B	109.5
C6—C5—H5	119.9	H16A—C16—H16B	109.5
C4—C5—H5	119.9	C15—C16—H16C	109.5
C5—C6—C7	120.8 (2)	H16A—C16—H16C	109.5
C5—C6—H6	119.6	H16B—C16—H16C	109.5
C7—C6—H6	119.6	C15—N1—C13	106.20 (19)
C6—C7—C12	121.6 (2)	C15—N1—Cu1	128.97 (16)
C6—C7—C8	119.0 (2)	C13—N1—Cu1	124.80 (15)
C12—C7—C8	119.3 (2)	C15—N2—C14	108.5 (2)
C9—C8—C3	123.2 (2)	C15—N2—H2	125.6
C9—C8—C7	117.7 (2)	C14—N2—H2	125.9
C3—C8—C7	119.1 (2)	C1—O1—Cu1	106.96 (15)
C10—C9—C8	121.1 (2)	N1—Cu1—N1^i^	180.00 (11)
C10—C9—H9	119.4	N1—Cu1—O1^i^	89.99 (7)
C8—C9—H9	119.4	N1^i^—Cu1—O1^i^	90.01 (7)
C9—C10—C11	121.0 (3)	N1—Cu1—O1	90.01 (7)
C9—C10—H10	119.5	N1^i^—Cu1—O1	89.99 (7)
C11—C10—H10	119.5	O1^i^—Cu1—O1	180.000 (1)
C12—C11—C10	119.8 (3)		
			
O2—C1—C2—C3	−127.3 (2)	C10—C11—C12—C7	−1.2 (4)
O1—C1—C2—C3	52.1 (3)	C6—C7—C12—C11	−179.3 (3)
C1—C2—C3—C4	−110.1 (3)	C8—C7—C12—C11	0.2 (4)
C1—C2—C3—C8	70.1 (3)	N1—C13—C14—N2	0.5 (3)
C8—C3—C4—C5	0.9 (4)	N2—C15—N1—C13	0.1 (3)
C2—C3—C4—C5	−178.9 (2)	C16—C15—N1—C13	179.9 (2)
C3—C4—C5—C6	0.5 (4)	N2—C15—N1—Cu1	−177.75 (15)
C4—C5—C6—C7	−1.2 (4)	C16—C15—N1—Cu1	2.1 (4)
C5—C6—C7—C12	179.9 (3)	C14—C13—N1—C15	−0.4 (3)
C5—C6—C7—C8	0.4 (4)	C14—C13—N1—Cu1	177.56 (17)
C4—C3—C8—C9	178.9 (2)	N1—C15—N2—C14	0.2 (3)
C2—C3—C8—C9	−1.3 (3)	C16—C15—N2—C14	−179.6 (2)
C4—C3—C8—C7	−1.6 (3)	C13—C14—N2—C15	−0.5 (3)
C2—C3—C8—C7	178.2 (2)	O2—C1—O1—Cu1	7.9 (3)
C6—C7—C8—C9	−179.5 (2)	C2—C1—O1—Cu1	−171.46 (15)
C12—C7—C8—C9	1.0 (3)	C15—N1—Cu1—O1^i^	−50.3 (2)
C6—C7—C8—C3	1.0 (3)	C13—N1—Cu1—O1^i^	132.28 (19)
C12—C7—C8—C3	−178.5 (2)	C15—N1—Cu1—O1	129.7 (2)
C3—C8—C9—C10	178.3 (2)	C13—N1—Cu1—O1	−47.72 (19)
C7—C8—C9—C10	−1.2 (3)	C1—O1—Cu1—N1	−95.44 (14)
C8—C9—C10—C11	0.2 (4)	C1—O1—Cu1—N1^i^	84.56 (14)
C9—C10—C11—C12	1.0 (4)		

Symmetry codes: (i) −*x*+1, −*y*, −*z*+2.

### Hydrogen-bond geometry (Å, °)

**Table:**

*D*—H···*A*	*D*—H	H···*A*	*D*···*A*	*D*—H···*A*
N2—H2···O2^ii^	0.86	1.97	2.775 (3)	155.

Symmetry codes: (ii) −*x*+1, −*y*+1, −*z*+2.
